# Can dogs sniff out cancer? Exploring the role of medical detection dogs in Hong Kong's early diagnosis strategies

**DOI:** 10.3389/fmed.2025.1644112

**Published:** 2025-11-24

**Authors:** Timothy Quan Lo, Bryden Chung Bun Tsui, Kei Shing Ng, Simon Ching Lam

**Affiliations:** 1 Queen Elizabeth School, Kowloon, Hong Kong SAR, China; 2 Cognitio College (Kowloon), Hong Kong, Hong Kong SAR, China; 3 Department of Diagnostic Radiology, Li Ka Shing Faculty of Medicine, The University of Hong Kong, Pok Fu Lam, Hong Kong SAR, China; 4 School of Nursing, Tung Wah College, Hong Kong, Hong Kong SAR, China

**Keywords:** medical detection dogs, cancer screening, volatile organic compounds, artificial intelligence analogy, public health

## Rationale and background

The advancement of artificial intelligence has substantially transformed modern healthcare, particularly in diagnostic fields such as medical imaging, genomics, and clinical decision support (1). Algorithms are now capable of detecting early signs of cancer from imaging scans (2), classifying pathology slides (3), and predicting disease risks with increasing precision (4). However, these technologies rely heavily on structured digital inputs and large volumes of labeled data. The collection, annotation, and processing of such data often require costly infrastructure, specialized software, and trained personnel, which can limit their accessibility in community settings and low-resource environments (5, 6).

This limitation invites us to consider whether natural biological systems, such as the canine sense of smell, could serve as a complementary approach. Dogs possess an extraordinarily powerful olfactory system that allows them to detect subtle chemical signatures, including volatile organic compounds present in human biological samples (7, 8). When trained using structured protocols, dogs have demonstrated the ability to identify diseases such as cancer (9–11), malaria (12), and most recently, COVID-19 (13). Recent studies and reviews further highlight their potential role in cancer detection, with growing evidence that trained dogs can discriminate cancer-related scent signatures from biological samples (10, 11). In several countries, trained detection dogs were successfully used to screen for COVID-19 infection in public settings, including airports and mass gatherings, with high reported sensitivity (96%) and specificity (95%) (13).

These findings suggest that trained dogs could offer an innovative, non-invasive method for population-based disease screening. Their ability to process complex scent information in real time makes them a valuable addition to the broader diagnostic toolkit, particularly in contexts where conventional testing may be logistically challenging or economically prohibitive.

## Local relevance in Hong Kong

Cancer is the leading cause of death in Hong Kong and accounts for about 25% of all registered fatalities in 2022 (14). Despite this considerable burden, cancer screening programs remain limited in scope and availability (15). At present, only a colorectal cancer screening program is offered to individuals between the ages of 50 and 75 (16). Other common cancers such as lung, liver, breast, and pancreatic cancers lack structured public screening initiatives. As a result, many patients are diagnosed at later stages when treatment is more complex and survival rates are reduced.

In this context, the need for innovative, accessible methods for early detection is growing. Hong Kong presents a unique opportunity to pilot new approaches due to its dense population (17), advanced healthcare infrastructure (18), and experience in public health operations. One promising direction may involve leveraging trained detection dogs. The use of detection dogs by the Hong Kong Customs and Excise Department to identify illegal drugs, firearms, and contraband goods has demonstrated their reliability in high-traffic, real-world settings (19). However, it is important to note that medical biodetection is considerably more complex, as volatile organic compound profiles associated with disease are more variable and subtle than contraband odors. Consequently, training techniques from law enforcement contexts cannot be directly applied and must be specifically adapted for medical detection. This established success in high-traffic environments such as airports and border control points demonstrates the reliability of detection dogs in complex, real-world scenarios. Nonetheless, hospitals and healthcare facilities represent different working environments that may require dogs with distinct temperaments and tailored training approaches. This established success in high-traffic environments such as airports and border control points highlights the feasibility of applying detection dogs for rapid, accurate screening in real-world healthcare settings (20). However, Hong Kong's unique environment, including high levels of urban air pollution and dense, overlapping scent profiles, may interfere with the accuracy of dogs' ability to detect cancer-related volatile organic compounds. Pilot studies should therefore carefully evaluate how these environmental factors affect canine performance and adapt training protocols to ensure reliable use in both community and clinical settings.

Building on their established use in public health during the COVID-19 pandemic, medically trained detection dogs could be further adapted to support early cancer detection initiatives. If trained to recognize scent patterns associated with cancer, these dogs could be integrated into community clinics or screening centers to support prescreening. In practice, this would most feasibly involve analyzing biological samples (e.g., breath, urine, or saliva) collected from individuals, rather than direct person-to-person screening, as the training and implementation constraints differ significantly between these approaches. This approach would require collaboration across sectors, including veterinary science, oncology, public health, and behavioral training but could serve as an effective supplement to conventional diagnostic tools.

## Evidence base: remarkable nose of the dog

**Figure 1:**
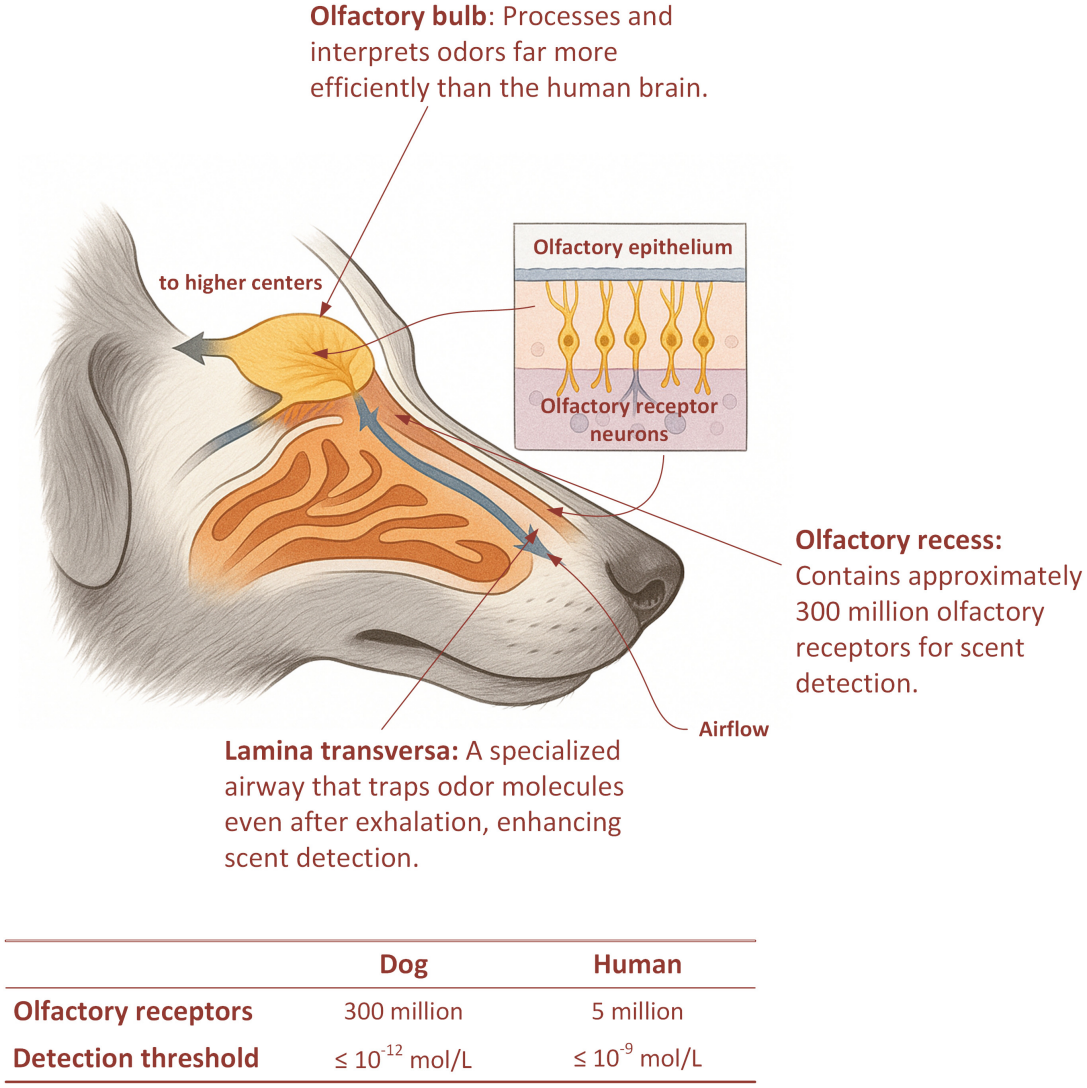
Anatomy and comparative capacity of the canine olfactory system. The olfactory epithelium lining the turbinates contains approximately 300 million olfactory receptors, enabling detection of minute concentrations of volatile organic compounds associated with disease. Airflow through the lamina transversa facilitates odor capture even during exhalation, while neural signals are processed in the olfactory bulb and relayed to higher brain centers. Inset: olfactory receptor neurons within the epithelium. Table: comparison of canine vs. human olfactory capacity (olfactory receptors = 300 million vs. = 5 million; representative detection thresholds ≤10^−12^ mol/L vs. ≤ 10^−9^ mol/L, depending on odorant (21, 22). Illustration created by the authors using Adobe Illustrator, adapted from anatomical descriptions in Craven et al. (21) and Hermanson and De Lahunta (23).

The dog (Figure 1) has one of the most advanced olfactory systems in the animal kingdom (24). Inside its nose are hundreds of millions of scent receptors, along with a highly developed olfactory bulb that processes chemical signals with exceptional precision. Dogs can detect minute amounts of volatile organic compounds (VOCs) released by the human body, including those linked to infections, metabolic changes, and even cancer (7, 25). However, a key limitation is that non-cancer factors such as infections, inflammation, diet, medications, or pollution may also alter VOC profiles (7, 26). Further research is needed to confirm whether dogs can consistently distinguish cancer-specific VOCs from these confounding sources. Nonetheless, their demonstrated ability to detect subtle volatile compounds provides the scientific rationale for training programs that harness this capacity for medical detection and early diagnosis.

For example, studies have shown that trained dogs can identify infectious diseases such as malaria and COVID-19 with high sensitivity and specificity (12, 27), and can also detect metabolic changes such as hypoglycemia in diabetes (28). These findings highlight the breadth of conditions detectable through canine olfaction and provide a stronger foundation for considering their application in medical contexts.

## Evidence base: training medical detection dogs

Just as artificial intelligence systems learn by analyzing large amounts of labeled data, medical detection dogs are trained through repeated exposure to biological samples collected from unique individuals. To ensure robust learning, these training sets should include samples from patients with confirmed cancer diagnoses (29), from individuals with other diseases that may mimic cancer-related scents, and from healthy controls drawn from similar environments as the cases and controls. Through positive reinforcement techniques, dogs learn to associate specific scent patterns, such as those released by cancer cells, with a reward. Importantly, these scent signatures may derive not only from the cancer cells themselves but also from the body's physiological and metabolic responses to the disease. Over time, they develop the ability to identify these scent markers with remarkable accuracy (30). However, one major challenge for wider adoption is the lack of standardized training protocols, sample collection methods (e.g., breath, urine, sweat), handling, and reward systems (29). Collaborative efforts across veterinary science, oncology, and public health are needed to develop rigorous, universally accepted guidelines. This training is similar to supervised learning in artificial intelligence, where a model improves its predictions by studying known examples. In both cases, structured training, validation, and continuous assessment are essential to ensure consistent performance in real-world conditions.

## Evidence base: dog training and its analogy to artificial intelligence

Training a medical detection dog shares important similarities with how artificial intelligence models are developed in healthcare. In supervised machine learning, an algorithm learns to recognize patterns in structured data by analyzing numerous labeled examples (31). These labels, such as “cancer” or “non-cancer”, allow the model to adjust and improve its predictions based on experience.

Similarly, detection dogs are exposed to biological samples from individuals with known medical conditions (30). Trainers present scent samples collected from patients diagnosed with cancer and reward the dog each time it correctly identifies a positive case. Through repetition and reinforcement, the dog learns to recognize scent profiles linked to disease. Crucially, effective biodetection training requires dogs not only to identify cancer-related VOC signatures but also to discriminate them from samples of individuals with other illnesses and from healthy controls, while generalizing across diverse individual scent backgrounds. The greater the diversity and quality of the training samples, the better the dog becomes at identifying new and unfamiliar cases, much like a data model that has been trained on a comprehensive dataset (29).

Evaluation plays a key role in both methods. Dogs are assessed with blind testing to measure their ability to identify disease without prior cues correctly, just as machine learning models are evaluated on separate validation sets. While dogs and artificial intelligence differ in form, they share a process of structured learning and performance measurement (Table 1). Dogs offer the advantage of mobility, fast response, and an ability to detect disease without relying on imaging or laboratory tools, which makes them suitable for use in community and field settings.

**Table 1:** Parallel features of medical detection dog training and artificial intelligence learning.

**Aspect**	**Detection dogs**	**Artificial intelligence (36)**
Learning method	Positive reinforcement with labeled scent samples	Supervised learning using labeled digital datasets
Input type	Biological samples (urine, breath, and sweat)	Structured data (images, text, and laboratory results)
Feedback loop	Reward for correct identification	Adjustment of model parameters using training data
Generalization ability	Improves with sample diversity and exposure	Improves with data size, balance, and training technique
Evaluation	Blind sample testing to measure sensitivity and specificity	Validation on separate test datasets
Advantages	Real-time, portable, low cost, and non-invasive	Scalable, consistent, and capable of processing large data volumes
Limitations	Requires maintenance and regular retraining	Requires infrastructure and large high-quality datasets

Both systems rely on exposure to labeled examples, repetition, and validation to develop pattern recognition capabilities. Dogs offer real-time, mobile screening with non-invasive sampling, whereas artificial intelligence provides scalable solutions for data-rich environments.

## Implementation pathway: strategy and relevance to Hong Kong

To harness the diagnostic potential of medical detection dogs, Hong Kong can adopt a flexible, community-centered implementation model. In countries such as the United Kingdom and Finland, trained dogs have been successfully deployed at healthcare facilities, transport hubs, and community screening events. Their agility and low operational burden make them particularly suitable for non-clinical settings (32, 33).

In Hong Kong, the Hospital Authority could integrate detection dogs into existing primary care services, including general outpatient clinics and public screening programs. A notable parallel is the colorectal cancer screening scheme, which has demonstrated that early detection substantially increases diagnosis rates compared with the general population (34). Hong Kong's Colorectal Cancer Screening Program reported a detection rate of 736.0 per 100,000 among screened participants, nearly double the incidence rate of 393.7 per 100,000 observed in the general population (34). A similar two-tiered strategy could be developed: canine prescreening to identify individuals with suspicious scent profiles, followed by targeted diagnostic imaging or laboratory testing for confirmation.

Importantly, the mobility of detection dogs opens further opportunities. These animals can be brought directly into community centers, residential care homes, and even individual residences. Nonetheless, training and maintaining a sufficient number of skilled detection dogs and handlers to cover Hong Kong's large population would pose logistical and financial challenges. For this reason, canine detection is best positioned as a prescreening tool or targeted intervention in high-risk or underserved groups, rather than a universal screening solution. Many old adults face mobility challenges and may not have easy access to magnetic resonance imaging or computed tomography scans. Canine screening could provide a rapid, noninvasive first step that identifies at-risk individuals who might otherwise go undiagnosed.

This strategy offers potential clinical and economic advantages. However, a comprehensive assessment of feasibility would need to consider costs such as initial and ongoing training, handler support, veterinary care, and the number of screenings each dog can realistically perform. Early identification facilitates timely intervention, reduces the need for advanced-stage treatment, and can decrease long-term healthcare costs. The scalable, portable nature of dog-based screening also enables outreach to underserved populations, which aligns with goals of public health equity and preventive care.

## Ethics, welfare, limitations, and conclusion

The integration of trained medical detection dogs into healthcare systems presents a unique opportunity to enhance early disease detection, particularly in settings where traditional diagnostics may be inaccessible, costly, or time consuming. In the era of artificial intelligence and advanced medicine, the remarkable sensory capabilities of dogs should not be overlooked. These capabilities are natural tools developed through evolution and successfully demonstrated in real-world applications, including the detection of cancers and infectious diseases. It is important to note that much of the current research on detection dogs has focused on training them to identify single cancer types, such as lung, breast, or pancreatic cancer (9, 25). However, recent studies suggest that trained dogs are capable of detecting a variety of cancers across different biological samples, indicating broader potential applications (10, 11). This highlights the need for cancer-specific training datasets and validation before broad application.

In Hong Kong, where an aging population and growing healthcare demands present substantial challenges, adopting a mobile, non-invasive, and cost-effective screening approach offers clear advantages. Whether positioned at outpatient clinics, community health centers, or brought to the homes of elderly individuals, detection dogs could serve as the first step in a tiered diagnostic model. Their role would be to identify individuals who may benefit from follow up investigations using imaging or laboratory testing. Importantly, practical and ethical aspects must be considered, including appropriate training methods, handler expertise, welfare safeguards (such as workload limits, rest periods, and veterinary oversight), and long-term sustainability. In addition, detection dogs should not be regarded merely as tools, but as sentient beings whose welfare must be safeguarded. Ethical safeguards should also include the consistent use of positive-reinforcement training, structured retirement planning, and transparent monitoring systems to ensure long-term welfare. These measures help maintain both animal wellbeing and public trust in the responsible deployment of medical detection dogs. Lessons learned from COVID-19 detection dog programs provide valuable insights into these challenges and their relevance for cancer detection (35).

The authors encourage public health authorities, researchers, and policymakers to explore pilot programs that assess the feasibility, accuracy, and value of canine-based screening in Hong Kong. Supporting this innovative, biologically grounded strategy can advance a new model of preventive healthcare, one that uses natural ability and modern science to improve access, equity, and outcomes for patients.
